# Supplementary material for: Determinants of chromosome-specific telomere lengths among 2573 All of Us participants
Source: Nat Commun. 2026 Mar 28;17:4579. doi: 10.1038/s41467-026-71172-x (PMC13195165; doi:10.1038/s41467-026-71172-x)
Supplement: Supplementary file 1 — Supplementary Information [file 41467_2026_71172_MOESM1_ESM.pdf]

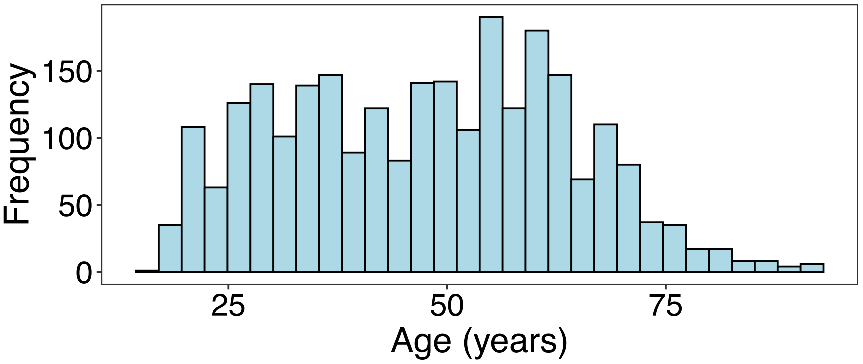

Supplementary Figure 1. Distribution of age (years) for All of Us participants (n=2,573)

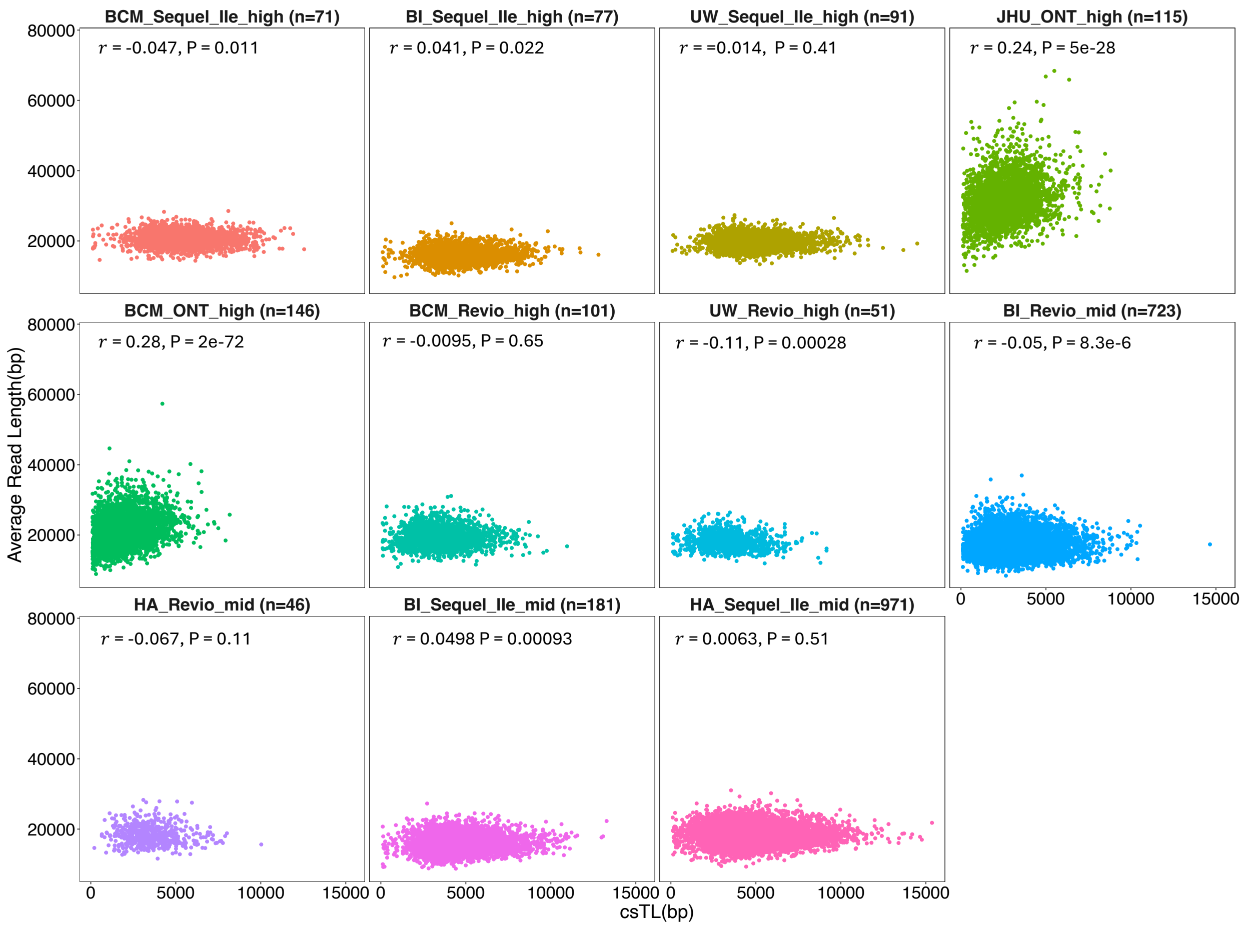

Supplementary Figure 2. Read length shows moderate correlations with csTLs in ONT samples only. Scatterplot showing the correlation between average read length and csTLs. Pearson correlation coefficient (r) and P-value displayed.

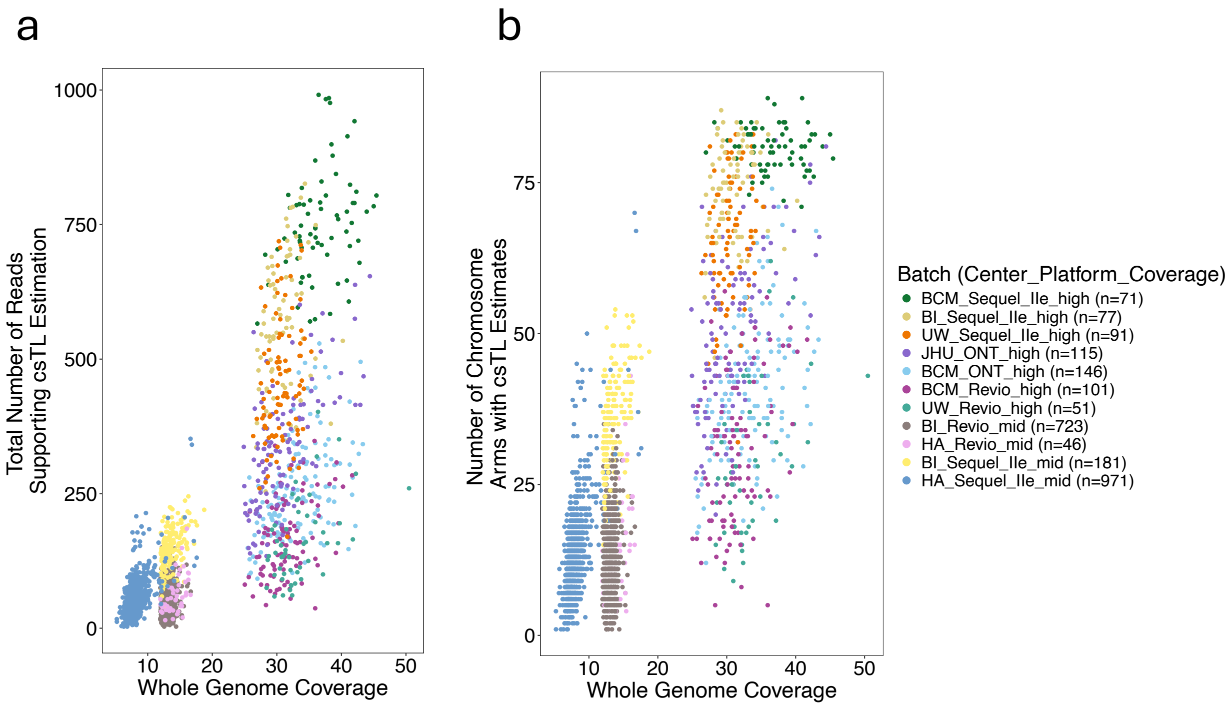

Supplementary Figure 3. Telogator2 performance shows inter-sample variability independent of whole-genome coverage. Scatterplots showing the relationship between whole-genome sequencing coverage and (a) total number of reads supporting csTL estimation. (b) the number of chromosome arms with csTL estimates. Point colors represent batch.

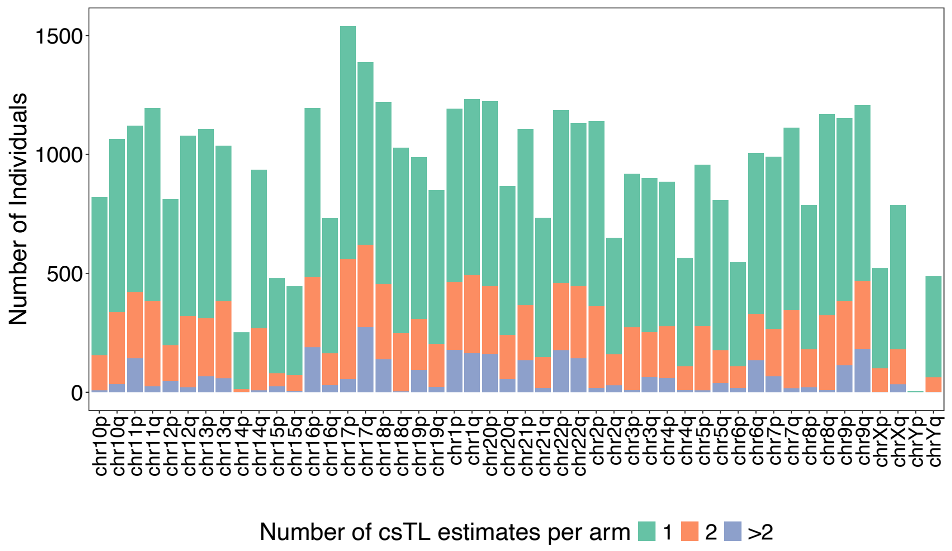

Supplementary Figure 4. Distribution of the number of TL estimates per chromosome arm across individuals. The x-axis shows each chromosome arm, and the y-axis indicates the number of individuals. Bars represent a stacked count of individuals with 1, 2, or >2 csTL estimates for that arm.

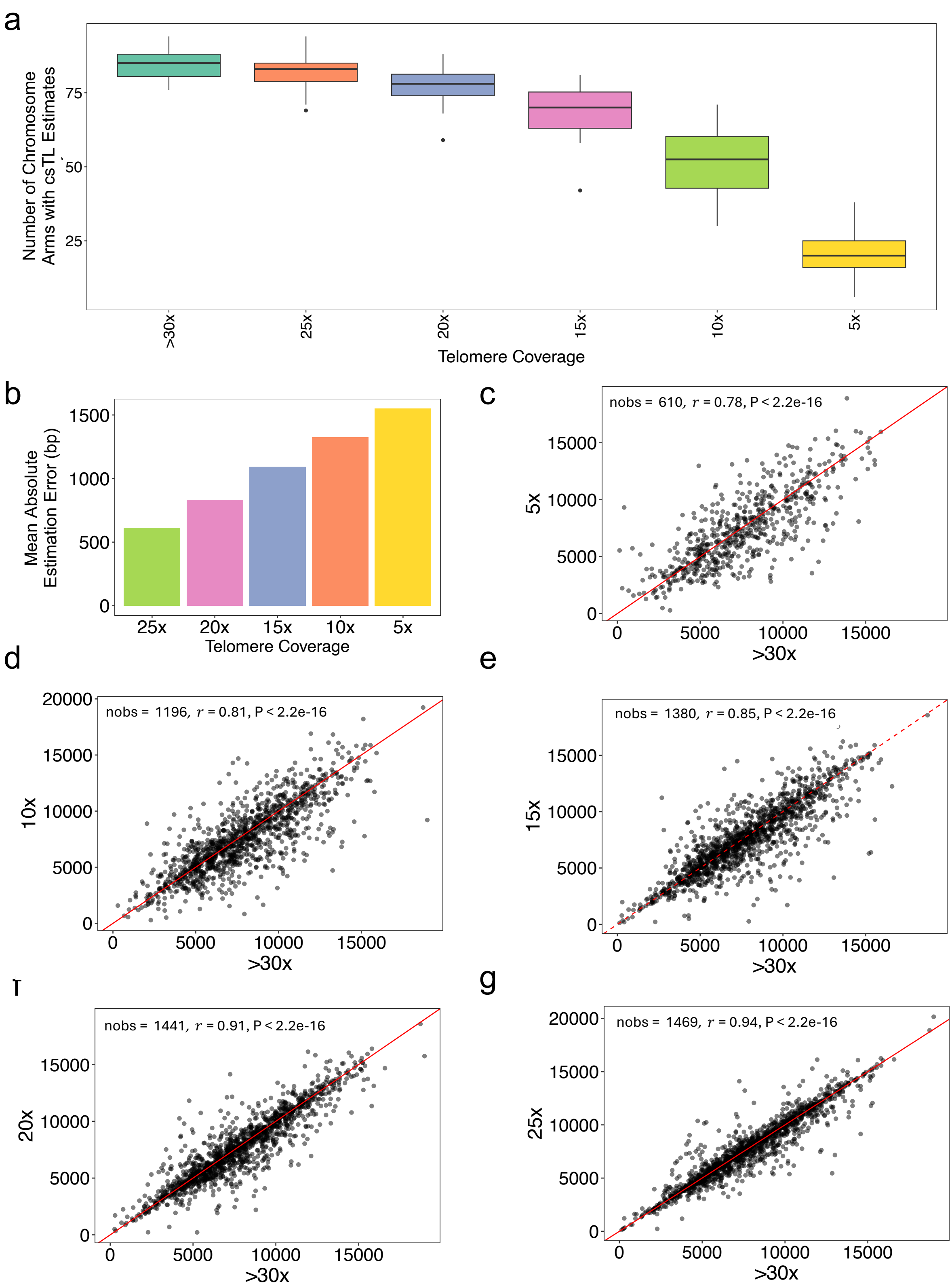

Supplementary Figure 5. Telogator2 performance across different telomeric coverage levels in HPRC samples (n = 36). (A) Box plot showing the number of chromosome arms with csTL estimates across telomere coverage levels. (B) Bar plot showing mean absolute estimation error between high-coverage (>30x) and down-sampled telomeric coverage levels. (C–G) Scatterplots comparing csTLs between >30× coverage and down-sampled telomeric coverage levels of (C) 5×, (D) 10×, (E) 15×, (F) 20×, and (G) 25×. Pearson correlation coefficient (*r*), P-value and the number of csTL observations (nobs) used to compute the correlation are displayed.

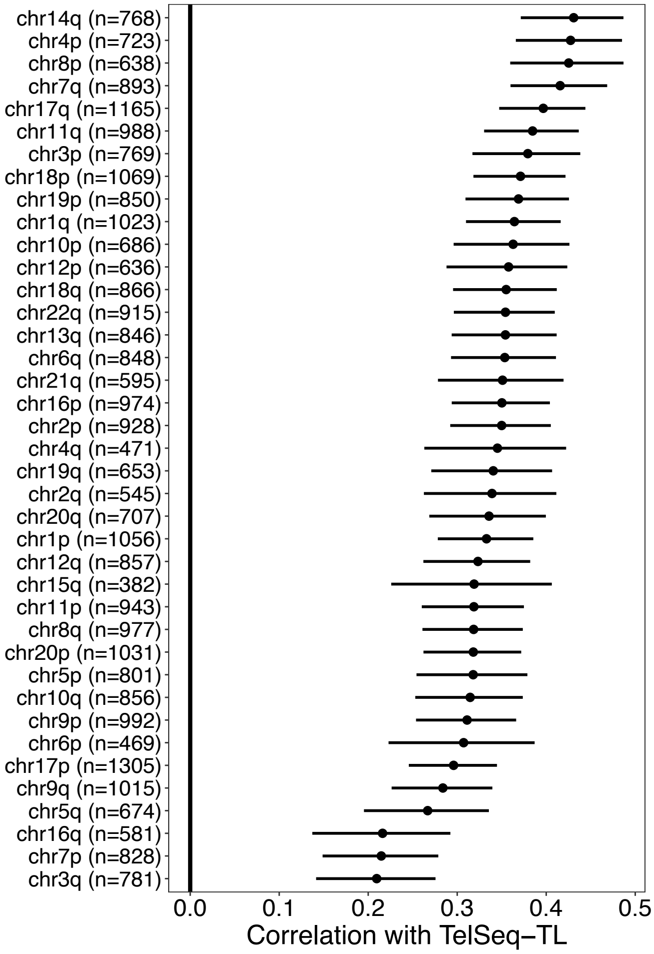

Supplementary Figure 6. csTL correlation with adjusted TelSeq-TL. Forest plot showing Pearson correlations (point) and 95% confidence intervals (horizontal bar) between principal component (PC) adjusted TelSeq-TL and csTL for all participants, excluding samples sequenced using Nanopore (ONT) (n=2,311).

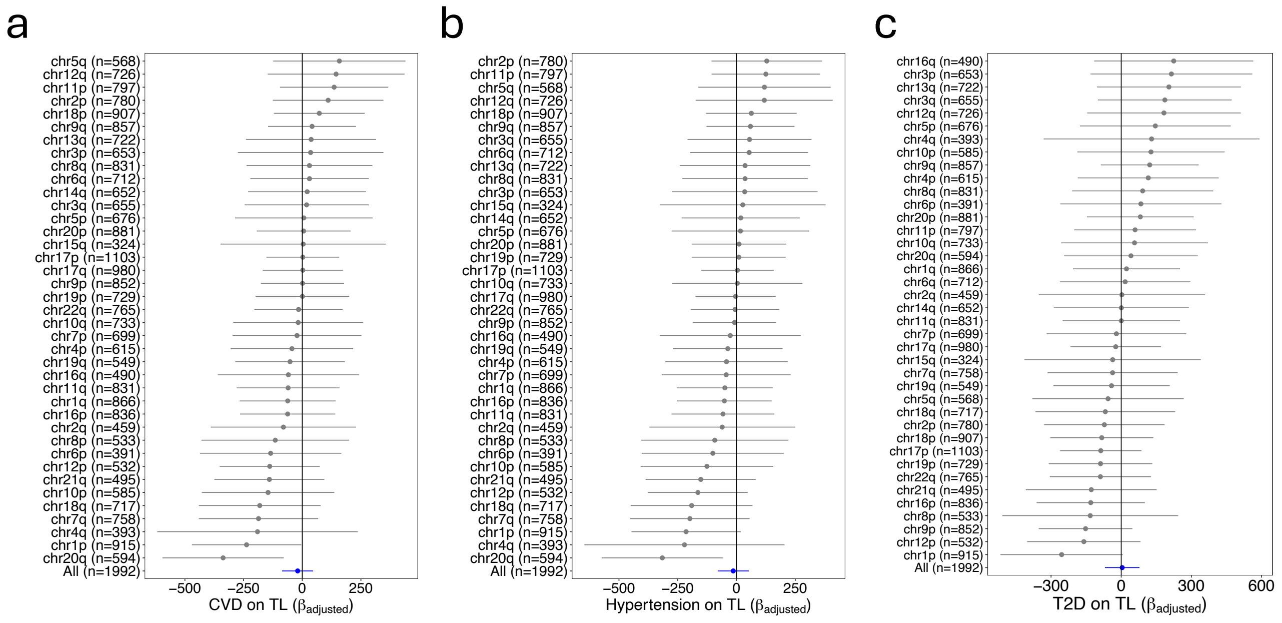

Supplementary Figure 7. Associations between chronic disease status and TL at individual chromosome arms and across all arms (in blue). Results are shown for (A) cardiovascular disease (844 cases, 1,148 controls), (B) hypertension (832 cases, 1,160 controls), and (C) type 2 diabetes (452 cases, 1,540 controls).

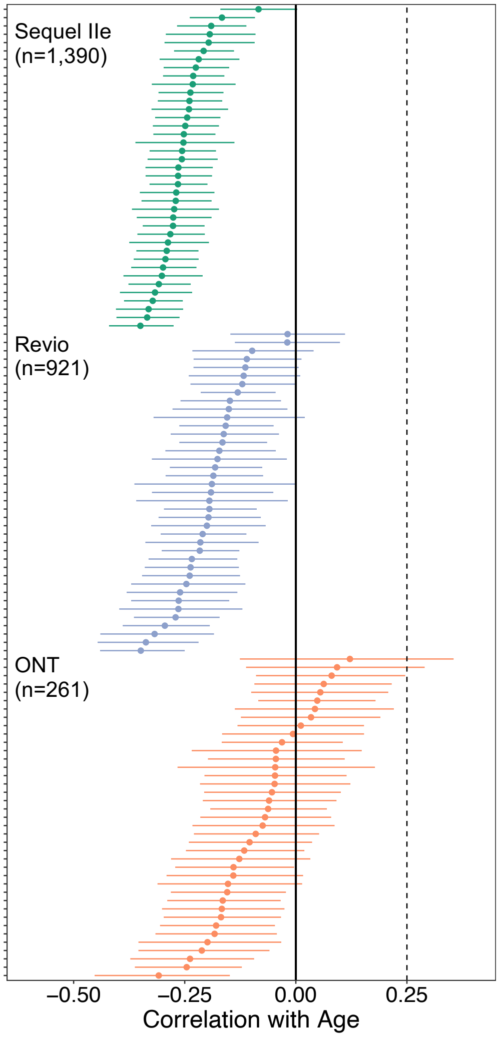

Supplementary Figure 8. ONT-derived csTLs show weaker associations with Age compared to PacBio-derived csTLs. Forest plot showing Pearson correlations (point) and 95% confidence intervals (horizontal bar) for the association between age (years) and csTLs for each sequencing platform.

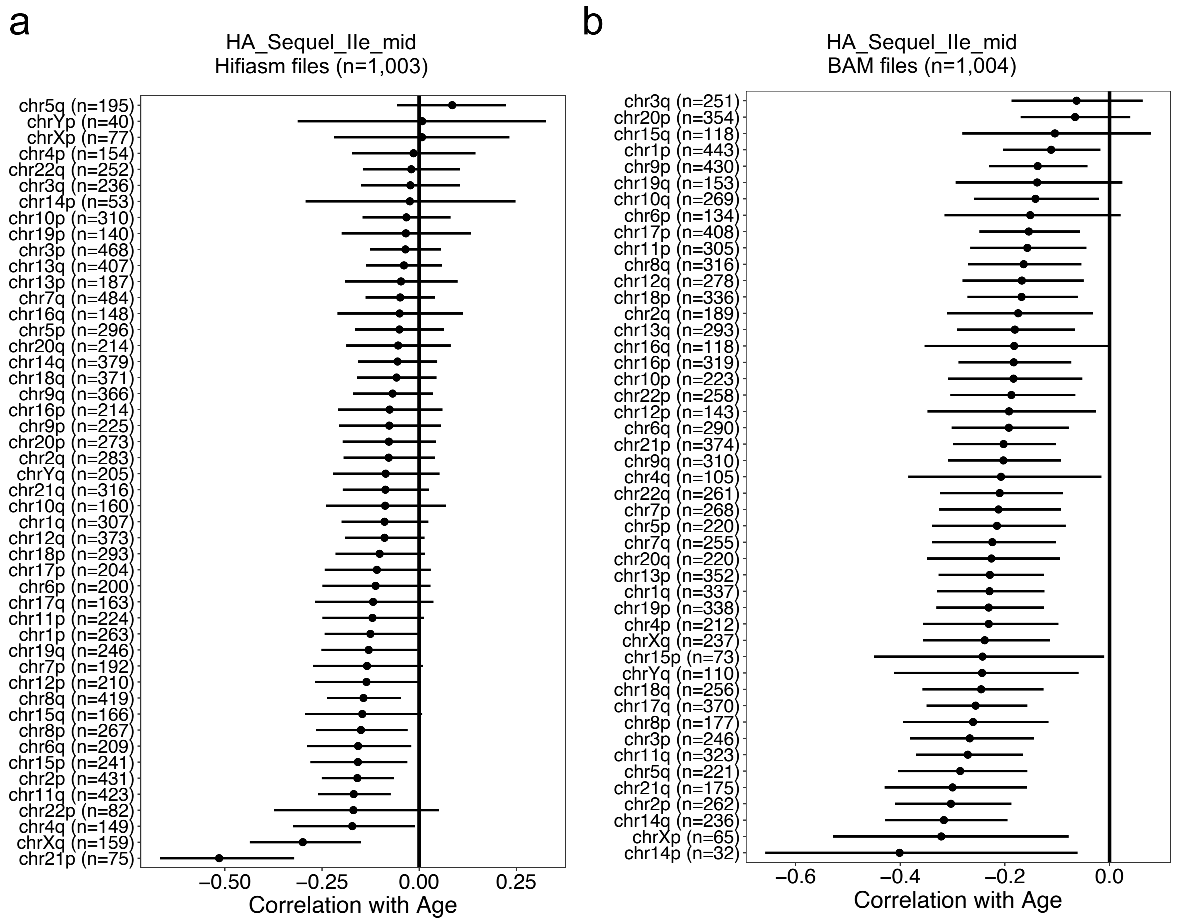

Supplementary Figure 9. BAM-based csTLs correlate more strongly with Age than hifiasm-based csTLs. Forest plot showing Pearson correlations (point) and 95% confidence intervals (horizontal bar) for the association between age (years) and csTLs for Hudson Alpha Institute (HA), Sequel IIe, mid-coverage samples (version 7 release), using (A) hifiasm files as input (n=1,003) and (B) BAM files as input (n=1,004)

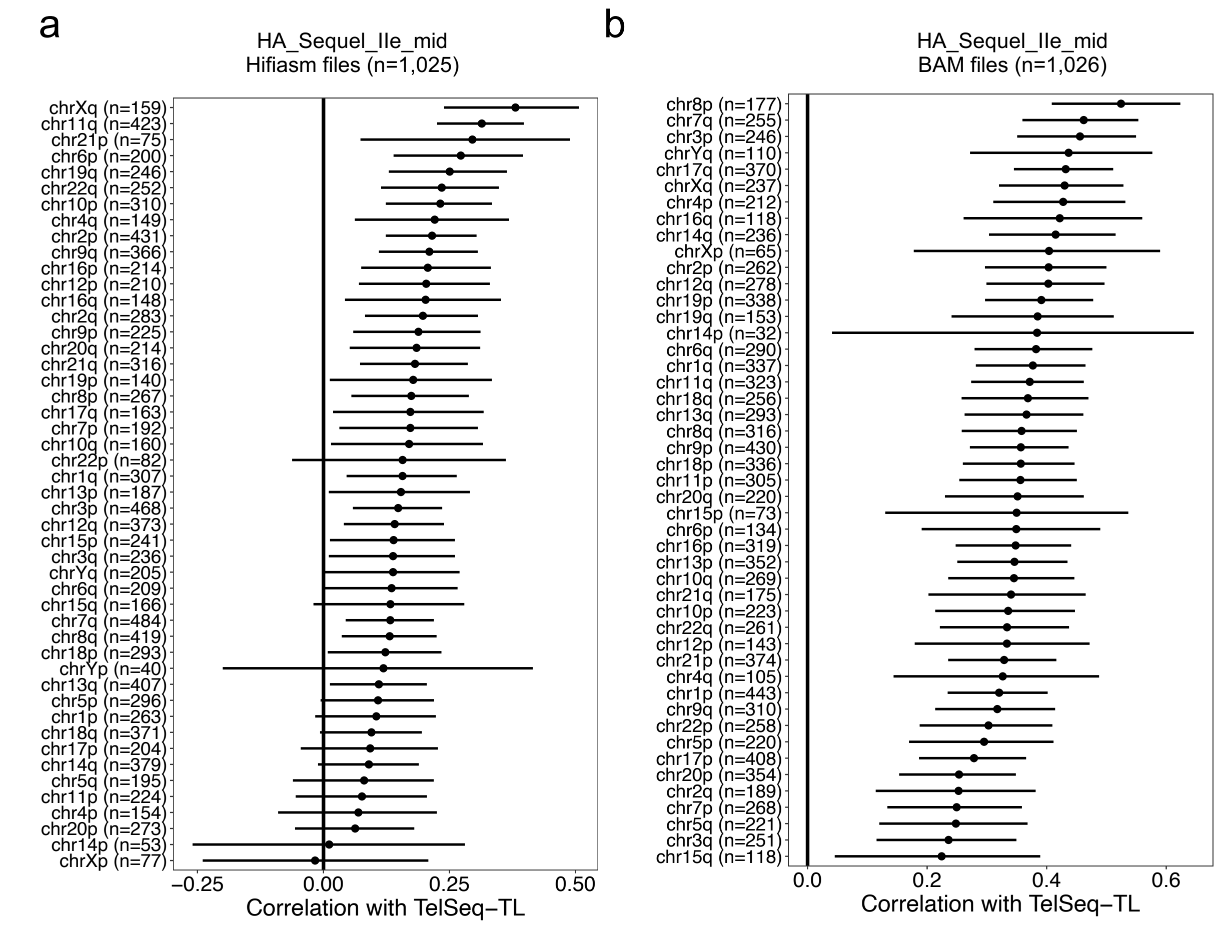

Supplementary Figure 10. BAM-based csTLs correlate more strongly with TelSeq-TL than hifiasm-based csTLs. Forest plot showing Pearson correlations and 95% confidence intervals for the association between TelSeq-TL and csTL estimates for Hudson Alpha Institute (HA), Sequel IIe, mid-coverage samples (version 7 release), using (A) hifiasm files as input for (n=1,025) and (B) BAM files as input (n=1,026)

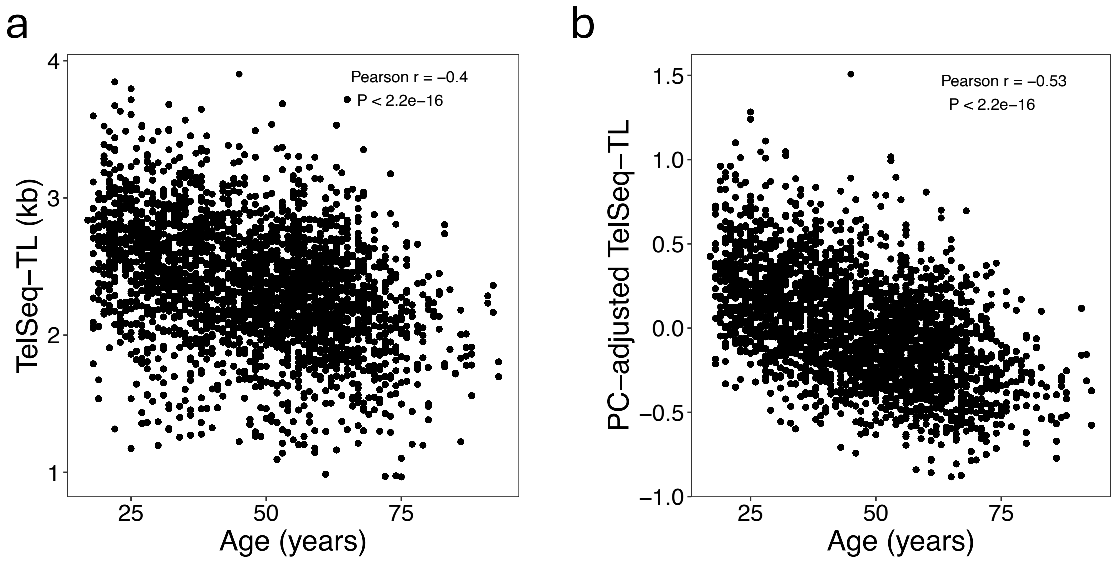

Supplementary Figure 11. Age is negatively correlated with TelSeq-TL. Scatterplot show the correlation between age (years) and **(A)** TelSeq-TL **(B)** principal component (PC) adjusted TelSeq-TL. Pearson correlation coefficient (*r*) and P-value (P) displayed.

Supplementary Table 1. Clinical codes extracted from the All of Us Researcher Workbench for disease identification.

	ICD-10	ICD-9
Cardiovascular Disease		
• Hypertension	I10, I11, I12, I13, I15	401, 402, 403, 404, 405
• Ischemic Heart Disease	I20, I21, I22, I25	410, 411, 413, 414
• Heart Failure	I50	428
	**Concept ID**	
Type 2 Diabetes	201826, 4008576, 4193704, 443732	
