# Supplementary material for: Determinants of chromosome-specific telomere lengths among 2573 All of Us participants
Source: Nat Commun. 2026 Mar 28;17:4579. doi: 10.1038/s41467-026-71172-x (PMC13195165; doi:10.1038/s41467-026-71172-x)
Supplement: Supplementary file 2 — Description of Additional Supplementary Files [file 41467_2026_71172_MOESM2_ESM.pdf]

Description of Additional Supplementary Files

Supplementary Data 1. Estimates of variation in csTL explained by fixed and random effects

Supplementary Data 2. Estimates of variation in csTL residuals explained by fixed and random effects after pre-adjusting for batch

Supplementary Data 3. Estimates of variation in csTL explained by fixed and random effects, with batch modeled as a random effect

Supplementary Data 4. Association between ancestry and TL for each chromosome arm

Supplementary Data 5. Association between sex and TL for each chromosome arm

Supplementary Data 6. Association between smoking status and TL for each chromosome arm

Supplementary Data 7. Association between age and TL for each chromosome arm

Supplementary Data 8. Interaction effect between fixed effect covariates and chromosome arm on TL variation

Supplementary Data 9. Association of chronic disease status with mean and shortest TL

Supplementary Data 10. Human Pangenome Reference Consortium (HPRC) samples analysed
