# Supplementary material for: Determinants of chromosome-specific telomere lengths among 2573 All of Us participants
Source: Nat Commun. 2026 Mar 28;17:4579. doi: 10.1038/s41467-026-71172-x (PMC13195165; doi:10.1038/s41467-026-71172-x)
Supplement: Supplementary file 4 — Reporting Summary [file 41467_2026_71172_MOESM4_ESM.pdf]

nature portfolio

Corresponding author(s): Niyati Jain
Last updated by author(s): Feb 27, 2026

Reporting Summary

Nature Portfolio wishes to improve the reproducibility of the work that we publish. This form provides structure for consistency and transparency in reporting. For further information on Nature Portfolio policies, see our Editorial Policies and the Editorial Policy Checklist.

Statistics

For all statistical analyses, confirm that the following items are present in the figure legend, table legend, main text, or Methods section.

n/a	Confirmed	
☐	☒	The exact sample size (n) for each experimental group/condition, given as a discrete number and unit of measurement
☐	☒	A statement on whether measurements were taken from distinct samples or whether the same sample was measured repeatedly
☐	☒	The statistical test(s) used AND whether they are one- or two-sided<br>*Only common tests should be described solely by name; describe more complex techniques in the Methods section.*
☐	☒	A description of all covariates tested
☐	☒	A description of any assumptions or corrections, such as tests of normality and adjustment for multiple comparisons
☐	☒	A full description of the statistical parameters including central tendency (e.g. means) or other basic estimates (e.g. regression coefficient) AND variation (e.g. standard deviation) or associated estimates of uncertainty (e.g. confidence intervals)
☐	☒	For null hypothesis testing, the test statistic (e.g. F, t, r) with confidence intervals, effect sizes, degrees of freedom and P value noted<br>*Give P values as exact values whenever suitable.*
☒	☐	For Bayesian analysis, information on the choice of priors and Markov chain Monte Carlo settings
☒	☐	For hierarchical and complex designs, identification of the appropriate level for tests and full reporting of outcomes
☐	☒	Estimates of effect sizes (e.g. Cohen's d, Pearson's r), indicating how they were calculated

Our web collection on statistics for biologists contains articles on many of the points above.

Software and code

Policy information about availability of computer code

Data collection

No software was employed for data collection

Data analysis

Chromosome-specific telomere length estimation:
Docker container for Telogator2 (niyatij/telogator2_image), Telogator2 (https://github.com/zstephens/telogator2)

Average telomere length estimation:
Docker container for TelSeq (jweinstk/telseq), TelSeq (https://github.com/zd1/telseq)
Docker container for Mosdepth zlskidmore/mosdepth:latest
NGS-PCA (https://github.com/PankratzLab/NGS-PCA)

R/4.4.0 packages (Jupyter Notebook):
lubridate
MuMln
lme4
Tidyverse (ggplot2, dplyr)
data.table
SQL embedded within Python (Jupyter Notebook) for All of Us participant characteristics and EHR data queries

For manuscripts utilizing custom algorithms or software that are central to the research but not yet described in published literature, software must be made available to editors and reviewers. We strongly encourage code deposition in a community repository (e.g. GitHub). See the Nature Portfolio guidelines for submitting code & software for further information.

Data

Policy information about availability of data

All manuscripts must include a data availability statement. This statement should provide the following information, where applicable:

- Accession codes, unique identifiers, or web links for publicly available datasets
- A description of any restrictions on data availability
- For clinical datasets or third party data, please ensure that the statement adheres to our policy

The data generated in this study, including the chromosome-specific telomere lengths from Telogator2 and TelSeq telomere lengths, have been deposited in the community workspace titled Jain_et_al_csTL_Nature_Comm, accessed within the featured workspace collection of the All of Us Researcher Workbench (https://support.researchallofus.org/hc/en-us/articles/360059633052-Featured-Workspaces). Access to this data is available to researchers affiliated with institutions that have signed a Data Use agreement with the All of Us Research program and who have obtained controlled tier access (https://www.researchallofus.org/register/). Supplementary Data 10 includes the list of Human Pangenome Reference Consortium samples analyzed, along with links for accessing the data.

Research involving human participants, their data, or biological material

Policy information about studies with human participants or human data. See also policy information about sex, gender (identity/presentation), and sexual orientation and race, ethnicity and racism.

Reporting on sex and gender	Sex was extracted from the "sex_at_birth" field under the All of Us Demographics concept set.
Reporting on race, ethnicity, or other socially relevant groupings	Ancestry information was derived from the ancestry prediction file provided by All of Us. It is reported as European, African, Admixed American, Middle Eastern, South Asian, East Asian. In the paper, Middle Eastern, South Asian, East Asian, are collapsed to a single category called 'Other'.
Population characteristics	Detailed in Table 1 ('Characteristics of All of Us participants included in csTL analysis using lrWGS data.') and Methods 'AoU cohort'. The study comprised of 2,573 individuals.
Recruitment	This study used de-identified data from the All of Us Research Program.
Ethics oversight	Informed consent was obtained by All of Us from all participants, and the All of Us Research Program protocol was by the NIH All of Us Institutional Review Board.

Note that full information on the approval of the study protocol must also be provided in the manuscript.

Field-specific reporting

Please select the one below that is the best fit for your research. If you are not sure, read the appropriate sections before making your selection.

☒ Life sciences ☐ Behavioural & social sciences ☐ Ecological, evolutionary & environmental sciences

For a reference copy of the document with all sections, see nature.com/documents/nr-reporting-summary-flat.pdf

Life sciences study design

All studies must disclose on these points even when the disclosure is negative.

Sample size	Sample sizes was chosen from the largest data available for individuals with long-read sequencing data and covariate data (age, sex, BMI, smoking status, and ancestry). Detailed in Methods under the sub-header 'AoU cohort'.
Data exclusions	Data were excluded when complete covariate information (age, sex, BMI, smoking status, and ancestry) was unavailable. For certain downstream analyses, samples sequenced using Nanopore were excluded, as we observed limited reliability for chromosome-specific telomere length estimation in our dataset.
Replication	NA
Randomization	No experimental vs control groups in this study.
Blinding	No experimental vs control groups in this study.

Reporting for specific materials, systems and methods

We require information from authors about some types of materials, experimental systems and methods used in many studies. Here, indicate whether each material, system or method listed is relevant to your study. If you are not sure if a list item applies to your research, read the appropriate section before selecting a response.

Materials & experimental systems

n/a	Involved in the study
☒	☐ Antibodies
☒	☐ Eukaryotic cell lines
☒	☐ Palaeontology and archaeology
☒	☐ Animals and other organisms
☒	☐ Clinical data
☒	☐ Dual use research of concern
☒	☐ Plants

Methods

n/a	Involved in the study
☒	☐ ChIP-seq
☒	☐ Flow cytometry
☒	☐ MRI-based neuroimaging

Plants

Seed stocks	NA
Novel plant genotypes	NA
Authentication	NA
